# Implementation of a high complexity nursing service in pediatric oncology

**DOI:** 10.1590/0034-7167-2023-0495

**Published:** 2025-03-10

**Authors:** Hanna Carolina Neto Cavalcanti, Tânia Cristina Franco Santos, Antonio José de Almeida, Camila Pureza Guimarães Silva, Marianne Cardoso Batallha, Elaine Lázaro Alcántara

**Affiliations:** I Universidade Federal do Rio de Janeiro. Rio de Janeiro, Rio de Janeiro, Brazil; II Universidad Catolica Santo Toribio de Mogrovejo. Chiclayo, Peru

**Keywords:** Nursing Service, Hospital, Oncology Nursing, Hospitals, Pediatric, History of Nursing, Nursing, Servicio de Enfermería en Hospital, Enfermería Oncológica, Hospitales Pediátricos, Historia de la Enfermería, Enfermería

## Abstract

**Objective:**

to analyze how the process of implementing an onco-hematology nursing service at a pediatric hospital in Rio de Janeiro took place.

**Methods:**

a historical, qualitative study. Sources included written and oral documents, produced through semi-structured interviews, carried out between August 2022 and February 2023 with nurses who worked on implementing the service, represented by 40 minutes, nine care protocols and eight reports from the hospital’s internal website.

**Results:**

nursing staff reorganization and investment in professional improvement, through courses and graduate courses, culminated in the establishment of protocols, committees and nursing indicators, which enabled the implementation of a service with quality and safety.

**Final considerations:**

the implementation of a nursing service in pediatric onco-hematology was successful and, while highlighting the importance of nursing, also delivered quality service to society, strengthening the Brazilian Health System.

## RESUMEN

**Objetivo:**

analizar cómo se desarrolló el proceso de implementación del servicio de enfermería de oncohematología en un hospital pediátrico de Río de Janeiro.

**Métodos:**

estudio histórico, cualitativo. Las fuentes incluyeron documentos escritos y orales, elaborados a partir de entrevistas semiestructuradas, realizadas entre agosto de 2022 y febrero de 2023 con enfermeros que trabajaron en la implementación del servicio, representados por 40 minutos, nueve protocolos de atención y ocho informes del sitio web interno del hospital.

**Resultados:**

la reorganización del equipo de enfermería y la inversión en superación profesional, a través de cursos y posgrados, culminaron en el establecimiento de protocolos, comités e indicadores de enfermería, que permitieron implementar un servicio con calidad y seguridad.

**Consideraciones finales:**

la implementación del servicio de enfermería en oncohematología pediátrica fue exitosa y, al tiempo que destacó la importancia de la enfermería, también entregó un servicio de calidad a la sociedad, fortaleciendo el Sistema Único de Salud.

## INTRODUCTION

Cancer is a disease with a long history dating back to Egyptian medical practices. From its emergence to the present day, it has been a devastating diagnosis, without a cure and multi-etiological, which has caused the need to approach it from different perspectives, especially when we talk about childhood cancer, whose social and family impact transcends the possibility of its acceptance^(1)^. This is an extremely difficult moment, which imposes an abrupt change and alters children’s daily life during treatment, especially with regard to distancing from emotional relationships^(2,3)^.

Cancer is the disease that kills the most children and adolescents worldwide. It is estimated that there are around 430,000 new cases per year in children and adolescents aged 0 to 19 years. The main diagnoses are leukemia, central nervous system tumors and lymphomas. In countries where the Human Development Index (HDI) is low or medium, the obstacle is to better use the financial resources allocated to health to improve prevention and early diagnosis so that cancer control is more effective.

Thus, 70% of new cases are concentrated in these countries, which clearly raises the need to implement public policies that can diagnose cancers early and with quality, that have adequate medications for treatment and that have a qualified staff to deal with the nuances that cancer has. The specificity of care in pediatric oncology demonstrates the need for greater attention to public policies and plans for cancer control and treatment. Many challenges in the field of policy need to be overcome to reach the level of care and service found in developed countries, including rapid and quality access to diagnosis and treatment, accessibility to medications and qualified staff. The availability of this triad is one of the greatest reasons why the survival rate in developed and developing countries is so discrepant when compared to the rate in underdeveloped countries^(4,5)^.

Facing cancer diagnosis in children has always been a governmental, social and political challenge, since its impact on the family implies a radical change in their life, their history and their time, aspects that nurses have been able to identify when conducting reconfiguration processes in caring for this type of patient, using education strategies, case management and the search for equity aligned with the social and political context of each era^(6)^.

Law 12732 of November 22, 2012 ensures that cancer patients have the right to begin oncological treatment within the Brazilian Health System (SUS – *Sistema Único de Saúde*) up to 60 days after the diagnosis has been included in their medical record. That same year, it was estimated that 11,530 children and adolescents aged 0 to 19 would be diagnosed with cancer, totaling around 3% of the total cases predicted for that year^(7)^.

In 2012, Rio de Janeiro had centers specialized in oncological treatment, but there was no state unit exclusively for pediatric onco-hematological treatment. In this context, there was a partnership between the State Government and the Social Health Organization (SHO) *Instituto D’Or de Gestão de Saúde Pública* to create and manage a state unit that offered this type of care. Thus, in March 2013, the *Hospital Estadual da Criança* was opened in Rio de Janeiro, in Vila Valqueire, classified as medium-sized, with medium and high complexity procedures^(8)^.

Before the opening of the *Hospital Estadual da Criança*, the space housed another private hospital, which assisted non-cancer patients of different age groups. With the contract with the State to open a new hospital with different characteristics from the previous one, it was necessary to readjust the nursing staff to meet the new demand.

In this way, around 70% of the nursing staff was relocated to other hospitals, compatible with the characteristics of the hospital that was in the process of closing its activities; 15% had their contracts terminated; and 15% of the nursing staff was absorbed by the new administration. Of these, the majority were nurses who held leadership positions, such as nursing coordinators.

Measures were necessary to reorganize nursing management and coordination, in order to handle the implementation of a nursing service in pediatric onco-hematology and care management, the specificities of which were unknown to most of the staff. Such measures were urgent and necessary for nursing to occupy leadership spaces.

In this context, the nurses who remained at the hospital began recruiting nurses for the new hospital space at the same time that they invested in formulating strategies aimed at incorporating knowledge, with the aim of meeting the challenges imposed in a highly specialized scenario of child care.

The historical situation presented therefore raised the following investigative questions: how was the hospital’s nursing service arranged in the onco-hematology sector when it opened? What strategies do nurses undertake to occupy leadership positions in hospital spaces?

## OBJECTIVE

To analyze how the process of implementing an onco-hematology nursing service at a pediatric hospital in Rio de Janeiro took place.

## METHODS

### Ethical aspects

The study was conducted in accordance with national and international ethical guidelines, approved by the *Escola de Enfermagem Anna Nery*/*Universidade Federal do Rio de Janeiro* Research Ethics Committee, whose opinion is attached to this submission. Consent was obtained from all individuals involved in the study by personally signing the Informed Consent Form. Anonymity was guaranteed through coding of statements and data confidentiality, saving the data in one of the researchers’ Google Drive. The study was approved under Opinion 5,521,434 and Certificate of Presentation for Ethical Consideration (CAAE - *Certificado de Apresentação para Apreciação Ética*) 58705822.6.0000.5238 on July 11, 2022.

### Study design

This is a historical study, with a qualitative approach. For its development, the COnsolidated criteria for REporting Qualitative research (COREQ) checklist criteria were followed.

### Methodological procedures

The study’s direct historical sources are written and oral documents. Participant inclusion criteria comprised nurses who worked on the implementation of a nursing service in the time frame of the study in question, i.e., between 2013-2016, in which the initial milestone corresponds to the year in which the *Hospital Estadual da Criança* was opened and the nursing staff was composed, and the final milestone represents the year in which the hospital was accredited as a High Complexity Unit in Oncology (UNACON). The use of these documents in this study is justified on the understanding that they provide objective information regarding the phenomenon studied. For this purpose, it was necessary to triangulate written and oral documents with the context in accordance with a historical analysis.

### Study setting

The spatial scope of this study is the *Hospital Estadual da Criança*, a setting that demarcated the phenomenon studied. When it opened, it had ten beds in the Neonatal Intensive Care Unit (ICU), ten beds in the Pediatric ICU, 58 beds in the Inpatient Unit and eight chemotherapy chairs for outpatient treatment for oncology patients, serving children aged 0 to 19 years.

### Data sources

The written documents, located in the hospital’s Quality Office, consist of: 40 meeting minutes; nine care protocols; eight reports from the internal website, called “intranet”, which make references to nursing weeks during the time frame; and oncological indicators (numerical indicators, in absolute numbers or percentages, prepared by the medical and nursing coordination for presentation to the State Department of Health, as determined by the Term of Reference “Healthcare service Management at the *Hospital Estadual da Criança* – *Oncologia e Cirurgia*, in the State of Rio de Janeiro, by a private, non-profit entity, qualified as a Social Organization” with the objective of hiring a public non-profit entity to manage, operationalize and execute healthcare services at the *Hospital Estadual da Criança*”), which demonstrated the performance of 170 diagnoses of onco-hematological diseases and administration of 9,181 chemotherapies. Oral documents were produced through six semi-structured interviews, all with nurses who participated in the implementation of a nursing service at the *Hospital Estadual da Criança*. For this article, four interviews were used, and they are described here as C1, C3, C4 and C5.

The documentary *corpus* constitution took into account the adequacy criterion, consisting of six stages: 1) relevance: historical sources belong to the subject studied; 2) sufficiency: responding to all dimensions of the research problem; 3) exhaustiveness; 4) eligibility, without exclusions of sources, due to researcher limitations; 5) representativeness: represent the global universe; 6) homogeneity: they are organized separately, since each type of source (written and oral) has different treatment and analysis^(9,10)^.

### Data collection and organization

Data collection took place from August 1, 2022 to February 13, 2023. Six interviews were carried out, and four had excerpts presented here, in direct contribution to the study. The four study collaborators, presented here, were contacted personally, and meetings were scheduled and interview locations were arranged.

The interviews were transcribed and returned to collaborators in the form of written text for validation, in order to check the transposition of the oral text to written text. Indirect sources, consisting of articles from scientific journals produced on the subject, substantiated the analysis of findings.

### Data analysis

The findings from written and oral sources were organized, triangulated and analyzed according to the historical method^(11)^. Thus, the analysis focuses on three pillars: heuristics, in which the sources of information that will be necessary for historical analysis are collected; criticism, to assess the validity or otherwise of sources; and hermeneutics, which enables the investigation of data and information in light of research questions. That said, the study results come from the intercession of the analysis of what is contained in written and oral sources, the study raw material, with indirect sources, based on the political, economic, social and health contexts in which the phenomenon studied is inserted. Data reliability was achieved through external criticism to verify the veracity of sources, considering intrinsic and extrinsic factors, criticism and internal, to assess validity and coherence based on the source itself. It is important to point out that the legitimization of documentary sources is a fundamental task in historical research with a qualitative approach referring to the near past, with interviews being a valuable resource to guide the findings^(12)^.

## RESULTS

From October 2010 to October 2012, there were 3,000 hospitalizations of children due to neoplasms, per year, in the city of Rio de Janeiro alone. Of these, only 2,000 were admitted to hospitals specializing in this type of treatment^(13)^. This scarcity of specialized care led to the proposal by the *Instituto D’Or de Gestão de Saúde Pública* (SHO qualified in the State to manage pediatric hospitals) to hire a private entity by the State of Rio de Janeiro.

Therefore, in 2012, the *Instituto D’Or de Gestão de Saúde Pública* prepared its Term of Reference entitled “Healthcare service Management at the *Hospital Estadual da Criança* – *Oncologia e Cirurgia*, in the State of Rio de Janeiro, by a private, non-profit entity, qualified as a Social Organization” with the objective “of hiring a public non-profit entity to manage, operationalize and execute healthcare services at the *Hospital Estadual da Criança*”^(8)^.

In accordance with what is established in the Term of Reference, the hospital provided was intended for the:

Reception of SUS users, referred by the State Department of Health of Rio de Janeiro to carry out surgical procedures, onco-hematological clinical treatment and hospitalization in a ward and beds in an intensive and post-operative care unit^(8)^.

In this context, in 2012, with the preparation of the Term of Reference for management dispute and, later, in 2013, upon signing the management contract between the State of Rio de Janeiro and the *Instituto D’Or de Gestão de Saúde Pública* to manage a hospital that demands the care of a specific human population, it was necessary to recruit qualified professionals to deal with this population, which required highly specialized attention.

Bearing in mind that, before the opening of the *Hospital Estadual da Criança*, the building provided to the Department of Health housed another private hospital, which closed its activities in 2012 to continue with the restructuring to open the new institution, in this old hospital, there was already a contracted nursing staff that endorsed nursing care. This was part of the management contract for *Rede D’Or*, which managed the old hospital, responsible for providing care to the adult public. Therefore, there was a movement to choose, by coordinators, employees who would remain under the new management and those who would be dismissed or reassigned to other hospitals in the same management group. The following speeches constitute the ratification of this movement, in which, based on the answer to the question “How did you get into the *Hospital Estadual da Criança*?”, it is possible to ascertain the selection from two study collaborators:

*When there was a move to the Hospital da Criança, some people were sent away; others were transferred to other hospitals in the network* [Rede D’Or]. (C3)

*I already worked at the previous hospital, Hospital Rio de Janeiro, I was a nurse in the emergency room, I wasn’t going to stay at the hospital, the people who would stay had already been chosen*. (C4)

Highly specialized hospital care requires professionals with knowledge so that they can provide quality care, meeting the demands of peer competitors who occupy leadership positions in this space. In this context, it was necessary to search for nursing professionals who demonstrated the ability to assist a specific human group, which required onco-hematological care.

Therefore, the hiring of new professionals followed unique requirements, and, for this, it was determined that experience in pediatrics and oncology was necessary, in addition to proof of specific training courses. This strategy aimed to form a group with expertise to deal with the details of onco-hematology and strengthen the nursing service that was being formed, and this plot is confirmed by one of the interviewees:

*We gave preference to hiring people with at least six months of experience in pediatrics. We preferred to also hire nurses specialized in neonatal and pediatrics, some in oncology and with some training, such as a catheter insertion course*. (C1)

When it opened, on March 1, 2013, the hospital had 46 nurses, arranged as follows: a nursing manager; five coordinators; three daily nurses; a nurse from the Hospital Infection Control Committee; and two continuing education nurses. In addition to these 12 nurses, all day laborers, there were also four nursing supervisors and 30 nurses who worked on duty. In this hospital, a daily nurse is understood to be someone who works daily in coordinating patient care. This quantity initially met the demands, as the opening of the hospital sectors was gradual, as informed by one of the collaborators:

*It was gradual, as I said at the beginning, so that we could start to receive the first children within the profile, the specialty, and then start scheduling and then the opening of hospitalization units and CCU, because both the CCU and the neonatal unit started with half the number of beds*. (C1)

News articles at the time the *Hospital Estadual da Criança* opened pointed to investment in quality of care in pediatric oncology in Rio de Janeiro, because, until the hospital’s opening, only the Brazilian National Cancer Institute (INCA - *Instituto Nacional do Câncer*) performed surgeries to treat cancer in children in the public network^(14)^.

The 46 nurses initially hired were arranged among the sectors by the remaining nurses, who held a leadership position and who sought, in the newly opened hospital, to undertake strategies to obtain professional recognition. The nursing coordination worked with the objective of establishing protocols aimed at patient safety and developing practices based on substantiated scientific evidence and with a view to improving care^(15,16)^. Interviewees’ reports show that there has been a large investment since the hospital’s opening, especially in 2014, in the development of protocols that support nursing care.

Protocols can be understood as a measure to reduce risks linked to healthcare and, consequently, to reduce unnecessary harm that may be imposed on patients, thus increasing their safety. Therefore, it is a strategy that aims to prevent the occurrence of preventable damage and, when it is not possible to avoid it, minimize its negative effect on patients as much as possible. Therefore, it is essential to assess which attitudes carried out by healthcare professionals promote patient safety and protocols enable this assessment^(17,18)^.

Among the established protocols, one can see the adequacy to the reality of onco-hematological nursing care, highlighting the intervention protocol in the face of accidental spillage of antineoplastic chemotherapy drugs, nursing care protocol to prevent extravasation of antineoplastic agents and nursing consultation in the onco-hematology outpatient clinic. The report of one of the interviewees makes clear the understanding of the importance of protocols for safe care:

*We participated in the production of these new protocols with nursing management.* [...] *and we brought contributions as we studied and put together these protocols*. (C5)

In parallel with the elaboration of protocols, hospital committees were created, responsible for assessing the care service effectiveness and quality, in addition to discussing specific cases, preparing opinions, proposing improvement initiatives, bringing suggestions for improving the service and assessing and developing indicators that demonstrate quality of care. The members of these committees were nominated and appointed by the hospital’s general director and medical director. One of the most important committees, chaired by a nurse, was the Central Venous Catheter Committee, which aimed to regulate the hospital’s venous devices, provide statistical data and promote the nursing staff technical-scientific development.

During the interviews, study collaborators reported that, after rearrangement of positions and allocations of day labor nurses, in order to work on health education in an expanded manner, nursing coordinators had the goal of reproducing knowledge in onco-hematology, acquired by them in training, graduate courses and visits to specialized units, to the hospital’s nursing staff.

The strategies, in order to seek professional improvement, can be observed when, two months after the hospital opened, in May 2013, the nurses already held the first Nursing Week. The program reinforced the concern with addressing topics related to onco-hematology and the necessary care, such as biosafety in chemotherapy (taught by an INCA speaker), clinical complications in oncology (lecturer on the clinical oncology graduate course), nursing management in the chemotherapy sector (nurse speaker at the *Hospital Pedro Ernesto*). As can be seen, there was concern in bringing nurses from reference hospitals, such as INCA, and large hospitals, such as *Hospital Pedro Ernesto*, to give lectures.

In compliance with contractual obligations, regarding accountability, contained in the Term of Reference, the hospital needed to develop indicators that demonstrated the effectiveness and quality of the care provided. Therefore, nurses had to respond to the request to create such indicators. Among the indicators developed, assessed and monitored, related to onco-hematology, were the number of chemotherapies administered, number of vesicant chemotherapies, number of chemotherapy spills, number of chemotherapeutic drug extravasation and number of extravasation injuries. These indicators would directly reveal nursing care quality, because they are activities exclusive to these professionals. Although they are not carried out directly by the coordination or management, the results would reflect their management, as activities are based on protocols developed and training carried out by them. This achievement made nurses known and recognized by peers, competitors or superiors within the institution.

Furthermore, some of the media articles related to the hospital referred to the fact that the *Hospital Estadual da Criança* had received the title of accreditation by the National Accreditation Organization (ONA - *Organização Nacional de Acreditação*) since 2014, being certified with excellence since 2015. ONA is an entity that, since 1999, has worked towards the adoption, by healthcare institutions, of management and care that leads to improved patient care and safety.

Patient safety in hospital units encourages discussions at national and international levels. In view of this, the World Health Organization (WHO) created a program in 2004 called the “World Alliance for Patient Safety”, aiming at assessing patient safety in healthcare services. Patient safety is a public health issue that reverberates around the world and requires universal and effective plans. Insecurity and harm caused to patients are the main causes of global diseases, the main preventable causes of which are medication administration errors and unsafe practices^(19)^.

Furthermore, annually, 134 million adverse events occur in hospitals in low- or middle-income countries, resulting in 2.6 million deaths due to unsafe care^(20)^. Despite the alarming numbers involving low and middle income countries, countries considered developed and centers of the world, such as the United States of America and the United Kingdom, are not completely safe from errors related to unsafe care, reflecting a damaging incident every 35 seconds in the United Kingdom and being the third cause of death in the United States of America^(21)^.

In Brazil, discussions related to patient safety have been taking place since 2002, with the creation of the Brazilian Network of Sentinel Hospitals by the Brazilian National Health Regulatory Agency (ANVISA - *Agência Nacional de Vigilância Sanitária*), however, only in 2013, the Brazilian National Patient Safety Program (PSP -*Programa Nacional de Segurança do Paciente*) was established by the Ministry of Health, through Ordinance MO 529, with the central objective of contributing to qualification of healthcare^(22)^. Since the establishment of PSP until 2022, there have been 1,100,352 reported incidents, but it is believed that this number could still be higher due to underreporting^(23)^.

The potential for risk or harm to patients is inherent to the healthcare sector, and the continuous search to minimize harm is essential. The WHO reveals that, for care to be considered quality, there must be acceptability, equity, safety, efficiency and effectiveness^(24)^. It also recommends in its document entitled “Patient safety: making health care safer”^(25)^ that healthcare establishments develop their activities based on accreditation and evidence and scientific practices.

Therefore, based on the indicator data presented to the Department of Health of the State of Rio de Janeiro, through the incorporation of a certificate of national importance, such as accreditation by ONA, presentation of specific care parameters and after presenting human resources in line with established standards, technical conditions, exclusive infrastructure and equipment and demonstrating specialized care in pediatric oncology and oncological hematology for children and adolescents, the *Hospital Estadual da Criança* was able to be qualified as UNACON in 2016.

According to INCA (2016), in 2006, there were 285 health establishments qualified for oncology care linked to SUS, and only 71 were qualified for pediatric oncology, i.e., these places made up a total of less than 25%. In the Southeast region, there were 136 services qualified in oncology and 35 in pediatric oncology, of which only five were in the state of Rio de Janeiro. According to Ordinance 140 of 2014, the city of Rio de Janeiro had 13 establishments qualified for oncology, but establishments qualified for pediatric oncology and hematology treatment remained five, and only one federal institution was registered as UNACON exclusively for pediatrics^(13)^.

The opening of the *Hospital Estadual da Criança* in 2013 brought to the state of Rio de Janeiro the possibility of onco-hematological diagnosis and treatment and diagnosis of other hematological diseases with exclusive service for children and young people. The portion of this population that was often attended to in nonspecialized centers or mixed units, and that spent long periods in regulatory queues, was able to stop traveling and begin the procedures for diagnosis and treatment in a place with quality care and specificity in pediatric onco-hematology, with exclusive facilities, access to all medications and equipment necessary for diagnosis-therapies and human resources compatible with the specific care that this population requires.

Furthermore, the pent-up demand that existed in 2012 gained a hospital capable of absorbing and being able, to a certain extent, to reach the triad of developed countries for successful treatment, i.e., qualified staff, rapid and quality access to diagnoses and treatment and accessibility to medications. In its opening year, the institution diagnosed 46 onco-hematological diseases and performed 1,175 chemotherapy treatments; in 2014, there were 40 onco-hematological diseases and 3,083 chemotherapy treatments; in 2015, it offered 39 diagnoses and performed 2,389 chemotherapy treatments; and in the last year of the study, in 2016, 45 children and adolescents were diagnosed and 2,534 chemotherapies were administered.

Between 2013 and 2016, the *Hospital Estadual da Criança* provided 170 diagnoses of malignant onco-hematological diseases and 263 diagnoses of benign onco-hematological diseases, which required specialist follow-up. This means that 433 children and adolescents were able to receive specialized attention and care and were not exposed to institutions that do not have staff with expertise in the area. Furthermore, in the same period, 9,181 chemotherapy administrations were carried out.

With regard to chemotherapies, these are carried out exclusively by trained nurses who have the skills to deal with clinical complications that may occur during administrations and which require plans to be drawn up to minimize risks to patients. The management of these risks, individually and based on technical and scientific concepts, as well as the number of indicators generated from this care, which demonstrate good nursing care, such as no injuries due to extravasation of chemotherapy drugs throughout the entire period of time, provision of specific courses in the area and constant protocol evolution, were certainly conditions assessed for qualification of the *Hospital Estadual da Criança* as UNACON to be granted, as provided for in Ordinance 2,491 of December 28, 2016.

## DISCUSSION

In Rio de Janeiro, in 2012, there was a pent-up demand for children and adolescents in need of onco-hematological care, due to the lack of specialized hospitals for this type of care^(13)^. In order to manage this issue, there was a partnership between the State of Rio de Janeiro government and an SHO, *Instituto D’Or*, to open a public hospital that would absorb this clientele for treatment. Due to the investment, *Instituto D’Or* needed to acquire specialized labor to handle this specific service.

SHOs are entities that are associated with the third sector. Legally, they are non-profit entities, but with private characteristics. The third sector brings a management model that, despite supporting various social care demands, “offers itself as an alternative for transferring social responsibilities from the State. The government assumes the role of regulator and non-governmental institutions support and replace the State”^(26)^.

To develop universal, comprehensive and equitable care, principles of SUS, it is necessary to manage the institution efficiently and with quality. To achieve this, managers need to organize the service, in order to guarantee the nursing staff’s performance in a resolute, efficient manner, which provides satisfaction to customers and, at the same time, motivates and gives legality to its staff^(27,28)^.

Therefore, regarding the strategies to implement the nursing service in 2012, when the *Hospital Rio de Janeiro* was dissolved to reformulate what would become the *Hospital Estadual da Criança*, remaining nurses who remained in charge of structuring this new service overcame the lack of technical knowledge in pediatric onco-hematology through the incorporation of technical-scientific knowledge, necessary to maintain their positions within the institution. They sought this knowledge in high-standard institutions, recognized for their work in pediatric onco-hematology, in addition to participation in courses and graduate courses that would grant them legitimate certifications that could ratify the inclusion of this new knowledge, thus starting the effective movement of implementing the service.

This is an effective strategy required to increase the knowledge of these nurses. Study with 1,312 nurses in five hospitals in Iran, on the development of an accreditation model, showed that investment in professional development impacts the improvement of services provided and that these initiatives contribute to achieving accreditation, which happened to the aforementioned hospital through nurses’ search for their qualifications^(29)^.

After the search for this knowledge, qualified personnel were hired in terms of management, i.e., those with specialized knowledge compatible with the upcoming demands in this space. Thus, the requirements were to have experience in onco-hematology, pediatrics and present specific courses in the area - *sine qua non* condition for implementing the service. The qualification of these professionals hired to develop the service within the *Hospital Estadual da Criança* occurred through the replication of knowledge acquired by the remaining nurses through training and targeted practices.

A recent integrative review showed that nurses develop, in their professional practice, the role of educators, promoting continuing education, developing actions aimed at teaching selfcare to patients, in addition to offering training and contributing to disseminating quality culture. Moreover, it seeks knowledge through the development of research and discussion of clinical cases^(29)^. In this sense, the qualification of professionals at the *Hospital Estadual da Criança* was essential for the successful implementation of the service, and emerges as one of the essential functions of nurses in processes of change at work, aiming at better service provision. The research also highlights the importance of managers encouraging and allocating resources to carry out education and training actions in institutions.

The choice of nurses who would remain in the new hospital clearly shows that they were preserved in their positions due to the hospital managers’ recognition of their technical qualities, which would enable the development of their coordination through the search for training and service restructuring strategies. Therefore, they were given the task of training the nursing staff that would provide the basis for that new reality.

These nursing coordinators, given the authority they exercised in the old hospital, were considered to have professional competence to promote the recruitment of nurses who would join the new institution. This condition can also be explained due to the necessary compatibility between the demands of the new experience with those already acquired, which placed them on another level in front of managers.

In relation to the number of nursing professionals, it is worth highlighting that, between 2013 and 2018, there was an increase of 4.7 million in the world, however, despite this increase, there is still an estimated shortage of 5.9 million professionals. In line with this, the WHO published, in 2020, a report entitled “State of the World’s Nursing 2020: Investing in Education, Jobs and Leadership”^(30)^, which highlighted the fundamental role of nursing in promoting health and preventing diseases. Nursing is the largest category of healthcare professional worldwide, accounting for 59% of the healthcare workforce.

The aforementioned report also highlights that the majority of these nurses work in low- or middle-income countries and that they face significant challenges, including inadequate conditions to carry out their work. It also brings as a fundamental point the demand to invest in leadership so that nurses play an active role in decision-making and development of health strategies.

That said, it could be seen that *Hospital Estadual da Criança* nurses implemented a nursing service in line with health demands, thus ensuring the quality of the service provided and promoting the nursing workforce’s relevance in a highly specialized scenario.

The partnership with institutions already recognized nationally, in relation to the educational sector, transformed the strategies for developing the service through the incorporation of knowledge about the management of nursing care for pediatric patients who required specialized attention. Furthermore, nursing training allows processes to be carried out in accordance with standards, in order to guarantee patient safety through the education of these professionals, being a tool for continuity of safe care^(31)^.

A systematic review, carried out between 1999 and 2021, to synthesize and assess the cumulative effect of patient safety educational intervention for healthcare professionals in the hospital environment on their patient safety culture, showed that education programs for this purpose can improve safety culture among healthcare professionals and patients, which supports the strategies developed by nurses at the *Hospital Estadual da Criança* ^(32)^.

This search for the incorporation of knowledge by the hospital’s nursing staff, with the purpose of consolidating a quality pediatric onco-hematology nursing service, is in line with the WHO policy in the Global Action Plan for Patient Safety 2021-2030, in which no patient can be a victim of adverse events, and must always receive safe and respectful care in all places and stages of care^(33)^. Improving patient safety is a policy objective in healthcare worldwide^(34)^.

Furthermore, patient safety, which requires continuous assessments and maintenance of a continuing education culture, based on the completion of improvement courses, institutionalization of protocols, indicators and scientific nursing committees, brought enough feedback for the *Hospital Estadual da Criança* to be assessed and certified by ONA and for it to be granted accreditation for excellence^(35)^. This is because patient safety represents one of the greatest challenges for healthcare organizations in the 21^st^ century and, therefore, requires the provision of quality services^(35)^.

Recent research highlights that a safety culture can be established through discussions about errors, adverse events and their impacts on quality of care. They also highlight that training programs are necessary to create a patient participation culture in managing their own care and improvements in the ability to identify patient safety incidents^(36,37,38)^.

An integrative review on nurses’ performance in a hospital accreditation context pointed out that nurses are leading actors in the search for certification, as they have responsibilities in care, administration and education, which are fundamental to achieving quality seal. It also highlights that accreditation encourages continuing education practices through training healthcare professionals with the aim of promoting best practices and developing professional profiles^(39)^. In this regard, due to accreditation, nurses used their leadership skills, ability to convey knowledge and ease of teamwork, which contributes to the successful implementation.

In 2023, the *Hospital Estadual da Criança* completed its first decade of operation and, in celebration, held a symposium entitled “A new reality of Public Health in Rio de Janeiro”, demonstrating the result of ten years of work and return to society. With 56 active inpatient unit beds, ten Pediatric ICU beds and ten Neonatal ICU beds, having started its activities in 2013 with 46 nurses, reaching 101 in 2023, carried out more than 200 thousand outpatient consultations in all its medical specialties and more than 22 thousand chemotherapy administrations, carried out by nurses, for cancer patients, making it possible to deliver quality of life to society for these children and adolescents as well as social, economic and personal development.

Furthermore, it is clear that nursing is the backbone of SUS, and its work can be seen in systematized actions aimed at comprehensive care to society. Being among only a few public hospitals in Brazil certified by ONA, title that was achieved with the effective participation of nurses, brings to light the weight that the implementation and consolidation of a nursing service had beyond the institution, reaching layers of society that, at another time, might not have had at their disposal a public healthcare service with the standard and experiences developed. This entire context and the *sine qua non* collaboration of nursing service in pediatric onco-hematology culminated in the *Hospital Estadual da Criança* qualification as the first (state) UNACON exclusively for pediatrics in Rio de Janeiro.

### Study limitations

A limitation of this study was the agreement, on the part of some collaborators, sometimes to grant the interview, sometimes to be located, which led to the search through social networks and instant messaging applications. Therefore, there was a delay in being able to carry out the interviews and also a smaller number of employees than initially expected. Despite these limitations, the answers to the investigative questions were answered.

### Contribution to nursing, health or public policy

The study demonstrates that the insertion of nurses in highly specialized areas, with a nursing service based on robust technicalscientific knowledge and a safety culture disseminated through protocols, commissions and indicators, directly implies the quality of service, being recognized and ratified not only by peers, but also by nationally renowned institutions, such as ONA, in addition to being qualified as UNACON exclusively for pediatrics by the Ministry of Health. That said, it encourages the achievement of SUS principles and returns to society what is expected: a differentiated nursing service profile that expands its operations, in order to positively impact the population’s lives.

## FINAL CONSIDERATIONS

The venture of implementing and consolidating a pediatric onco-hematology service was successful and allowed the nursing staff to capitalize on social recognition of its importance in the hospital’s healthcare staff.

Nurses were key elements in this process, as they played a caring, managerial, educational and political role. Protocols, formulations of indicators, committees and scientific events, while providing safety to patients, were also of great value for the hospital to be qualified as a UNACON exclusively for pediatrics, granted by the Ministry of Health, thus achieving the proposal of delivering to society a service of technical, scientific and human quality, contributing to strengthening SUS.

## References

[1] López MM, Cardona AF. (2021). Historia del cáncer y el cáncer en la historia. Med.

[2] Castro LS, Silva LJ, Silva TP, Queiroz GOM, Souza SR, Noronha RDB. (2023). Families of Children with in Pediatric Oncology Emergency Services: unveiling meanings. Texto Contexto Enferm.

[3] Oliveira LS. (2021). Câncer infantil: o impacto do diagnóstico para a criança e familiares. REASE.

[4] Pires LJA. (2018). Child and Adolescent Cancer in Public Policies in the State of Rio de Janeiro, 2013-2021. RBC.

[5] Maraba RRB, Lima SFS, Bezerra DG, Lima AS, Reis RPR. (2019). The role of the nurse in front of a child hospitalized with cancer. BJSCR[Internet].

[6] Tovar Neira LA, Cacante Caballero JV. (2023). El cuidado de Enfermería en cáncer infantil: una mirada desde los patrones sociopolítico y emancipatorio. Investg Enferm Imagen Desarol [Internet].

[7] Ministério da Saúde (BR) (2012). Lei nº 12732 de 22 de novembro de 2012. Dispõe sobre o primeiro tratamento de paciente com neoplasia maligna comprovada e estabelece prazo para seu início[Internet].

[8] Governo do Estado do Rio de Janeiro (2012). Secretaria de Estado de Saúde. Termo de Referência: Gestão de Serviços de Saúde no Hospital Estadual da Criança – Oncologia e Cirurgia, no Estado do Rio de Janeiro, por entidade de direito privado, sem fins lucrativos, qualificada como Organização Social tendo como objeto a contratação de entidade pública sem fins lucrativos para gerir, operacionalizar e executar os serviços de saúde do Hospital Estadual da Criança – Oncologia e Cirurgia.

[9] Barros JA. (2020). A Fonte Histórica e seu lugar de produção.

[10] Barros JA. (2021). O uso dos conceitos: uma abordagem interdisciplinar.

[11] Padilha MI, Bellaguarda MLR, Nelson S, Maia ARC, Costa R. (2017). O uso das fontes na condução da pesquisa histórica. Texto Contexto Enferm.

[12] Carlos DJD, Bellaguarda MLR, Padilha MI. (2022). The document as a primary source in nursing and health studies: a reflection. Esc Anna Nery.

[13] Ministério da Saúde (BR) (2016). Instituto Nacional do Câncer José de Alencar. Incidência, mortalidade e morbidade hospitalar por câncer em crianças, adolescentes e adultos jovens no Brasil: informações dos registros de câncer e do sistema de mortalidade [Internet].

[14] Conselho Nacional de Secretários de Saúde (CONASS) (2013). Rio de Janeiro ganha Hospital Estadual da Criança [Internet].

[15] Mendes LA, Costa ACL, Silva DCZ, Simões DAS, Corrêa AR, Manzo BF. (2021). Adherence of the nursing team to patient safety actions in neonatal units. Rev Bras Enferm.

[16] Oliveira TGP, Diniz CG, Carvalho MPM, Corrêa AR, Rocha PK, Manzo BF. (2022). Involvement of companions in patient safety in pediatric and neonatal units: scope review. Rev Bras Enferm.

[17] Aranha GA, Cruz AC, Pedreira MLG. (2023). Medication reconciliation in pediatrics: a validation of instruments to prevent medication errors. Rev Bras Enferm.

[18] Brás CPC, Ferreira MMC, Figueiredo MCAB, Duarte JC. (2023). Patient safety culture in nurses’ clinical practice. Rev Latino-Am Enfermagem.

[19] World Health Organization (WHO) (2018). 71st World health assembly 2018 side event–summary: Global Action on Patient Safety for Achieving Effective Universal Health Coverage [Internet].

[20] World Health Organization (WHO) (2021). Plano de ação global para a segurança do paciente 2021-2030: Em busca da eliminação dos danos evitáveis nos cuidados de saúde[Internet].

[21] Metelski FK, Engel FD, Ferreira de Mello AL, Meirelles BH. (2023). A segurança do paciente e o erro sob a perspectiva do pensamento complexo: pesquisa documental. Physis: Rev Saúde Coletiva.

[22] Ministério da Saúde (BR) (2013). Portaria nº 529, de 1 de abril de 2013. Institui o Programa Nacional de Segurança do Paciente[Internet].

[23] Agência Nacional de Vigilância Sanitária (Anvisa) (2022). Boletim de Segurança do Paciente em Qualidade em Serviços de Saúde n° 29: incidentes relacionados à Assistência à Saúde – 2014 a 2022 [Internet].

[24] Oliveira JLC, Cervilheri AH, Haddad MCL, Magalhães AMM, Ribeiro MRR, Matsuda LM. (2020). Interface entre acreditação e segurança do paciente: perspectivas da equipe de enfermagem. Rev Esc Enferm USP.

[25] World Health Organization (WHO) (2017). Patient safety: making health care safer [Internet].

[26] Turino F, Fernandes LEM, Soares GB, Castro GB, Salles SM, Zaganelli JC (2022). Seguindo o dinheiro: análise dos repasses financeiros do Município do Rio de Janeiro para as Organizações Sociais de Saúde. Cad Saude Publica.

[27] Silva CPG, Aperibense PGGS, Almeida AJ, Santos TCF, Nelson S, Peres MAA. (2020). From in-service education to continuing education in a federal hospital. Esc Anna Nery.

[28] Berghetti L, Franciscatto LHG, Getelina CO. (2019). Formação do enfermeiro acerca do gerenciamento: entraves e perspectivas. RECOM.

[29] Mosadeghrad AM, Ghazanfari F. (2021). Developing a hospital accreditation model: a Delphi study. BMC Health Serv Res.

[30] World Health Organization (WHO) (2020). State of the World’s Nursing 2020: investing in education, jobs and leadership[Internet].

[31] Rodrigues TA, Amaral FMA, Hoffmann MA, Azevedo C, Ribeiro HCTC, Mata LRF. (2023). Factors associated with the safety culture of patients under dialysis in the context of the COVID-19 pandemic. Rev Bras Enferm.

[32] Agbar F, Zhang S, Wu Y, Mustafa M. (2023). Effect of patient safety education interventions on patient safety culture of health care professionals: systematic review and meta-analysis. Nurs Educ Pract.

[33] World Health Organization (WHO) (2021). Global patient safety action plan 2021–2030: towards eliminating avoidable harm in health care Geneva; 2021 [Internet].

[34] World Health Organization (WHO) (2020). Towards eliminating avoidable harm in health care: draft global patient safety action plan[Internet].

[35] Batista SA, Miclos PV, Amendola F, Bernardes A, Mohallem AGC. (2021). Authentic leadership, nurse job satisfaction and hospital accreditation: a study in a private hospital network. Rev Bras Enferm.

[36] Körner M, Dinius J, Ernstmann N, Heier L, Bergelt C, Hammer A. (2023). Effectiveness and feasibility of an interprofessional training program to improve patient safety: a cluster-randomized controlled pilot study. Front Psychol.

[37] Sahlström M, Partanen P, Azimirad M, Selander T, Turunen H. (2019). Patient participation in patient safety: an exploration of promoting factors. J. Nurs. Manag.

[38] Parente AN, Ferreira GRON, Cunha CLF, Ramos AMPC, Sá AMM, Haddad MCFL (2024). Educação permanente para qualidade e segurança do paciente em hospital acreditado. Acta Paul Enferm.

[39] Cunha SGS, Pereira Torres K, Gomes de Morais MH, Santos e Alves S, Guerra Siman A, Menezes Brito MJ. (2021). Atuação do enfermeiro no contexto da acreditação hospitalar: uma revisão integrativa. Enferm Actual Costa Rica.

