# Supplementary material for: Implementation of a high complexity nursing service in pediatric oncology
Source: Rev Bras Enferm. 2025 Mar 10;78(1):e20230495. doi: 10.1590/0034-7167-2023-0495 (PMC11893063; doi:10.1590/0034-7167-2023-0495)
Supplement: Translation

# Translation: Implantação de um serviço de enfermagem de alta complexidade em oncologia pediátrica

**DOI:** 10.1590/0034-7167-2023-0495pt

**Authors:** Hanna Carolina Neto Cavalcanti, Tânia Cristina Franco Santos, Antonio José de Almeida, Camila Pureza Guimarães Silva, Marianne Cardoso Batallha, Elaine Lázaro Alcántara

**Affiliations:** I Universidade Federal do Rio de Janeiro. Rio de Janeiro, Rio de Janeiro, Brasil; II Universidad Catolica Santo Toribio de Mogrovejo. Chiclayo, Peru

**Keywords:** Serviço Hospitalar de Enfermagem, Enfermagem Oncológica, Hospitais Pediátricos, História da Enfermagem, Enfermagem

## Abstract

**Objetivo:**

analisar como se deu o processo de implantação do serviço de enfermagem em onco-hematologia de um hospital pediátrico no Rio de Janeiro.

**Métodos:**

estudo histórico, qualitativo. Fontes incluíram documentos escritos e orais, produzidos por entrevistas semiestruturadas, realizadas entre agosto de 2022 e fevereiro de 2023 com enfermeiras que atuaram na implantação do serviço, representados por 40 atas, nove protocolos assistenciais e oito reportagens do site interno do hospital.

**Resultados:**

a reorganização da equipe de enfermagem e o investimento no aprimoramento profissional, por meio de cursos e pós-graduações, culminaram na instituição de protocolos, comissões e indicadores de enfermagem, os quais possibilitaram a implantação de um serviço com qualidade e segurança.

**Considerações finais:**

a implantação do serviço de enfermagem em onco-hematologia pediátrica foi exitosa e, ao tempo que evidenciou a importância da enfermagem, também entregou à sociedade um serviço de qualidade, fortalecendo o Sistema Único de Saúde.

## INTRODUÇÃO

O câncer é uma doença com uma longa história que remonta às práticas médicas egípcias. Do seu surgimento aos dias atuais, configurou-se um diagnóstico devastador, sem cura e multietiológico, o que causou a necessidade de abordá-lo sob diferentes óticas, sobretudo quando falamos sobre o câncer infantil, cujo impacto social e familiar transcende a possibilidade de sua aceitação^(1)^. Trata-se de um momento extremamente difícil, que impõe uma mudança abrupta e que altera o cotidiano da criança durante o tratamento, principalmente no tocante ao distanciamento das relações afetivas^(2,3)^.

O câncer é a doença que, mundialmente, mais mata crianças e adolescentes em todo o mundo. Estima-se que ocorrem cerca de 430 mil novos casos por ano em crianças e adolescentes de 0 a 19 anos de idade. Os principais diagnósticos são as leucemias, os tumores do sistema nervoso central e os linfomas. Em países em que o Índice de Desenvolvimento Humano (IDH) é baixo ou médio, o obstáculo é utilizar melhor os recursos financeiros deslocados para a saúde para melhorar a prevenção e diagnóstico precoce, para que o controle do câncer seja mais eficaz.

Nesses países, concentram-se 70% dos casos novos, o que, claramente, traz a necessidade de implementação de políticas públicas que possam diagnosticar os cânceres de forma precoce e com qualidade, que disponham de medicamentos adequados para o tratamento e que possuam uma equipe qualificada para lidar com as nuances que o câncer possui. A especificidade do cuidado em oncologia pediátrica demonstra a necessidade de maior atenção às políticas e planos públicos de controle e tratamento do câncer. Muitos desafios no campo da política precisam ser transpostos para se alcançar o nível de atenção e atendimento de países desenvolvidos, como acesso rápido e de qualidade a diagnóstico e tratamento, acessibilidade a medicamentos e equipe qualificada. A disponibilidade dessa tríade é uma das maiores razões para que a taxa de sobrevida em países desenvolvidos e em desenvolvimento seja tão discrepante quando comparada à taxa de países subdesenvolvidos^(4,5)^.

Enfrentar o diagnóstico de câncer em crianças sempre foi um desafio governamental, social e político, uma vez que seu impacto na família implica uma mudança radical em sua vida, sua história e seu tempo, aspectos que os enfermeiros têm conseguido identificar ao conduzir processos de reconfiguração no cuidado a esse tipo de paciente, fazendo uso de estratégias de educação, gestão de casos e busca de equidade alinhadas ao contexto social e político de cada época^(6)^.

A Lei n° 12.732, de 22 de novembro de 2012, assegura que os pacientes com câncer tenham direito ao início do tratamento oncológico no âmbito do Sistema Único de Saúde (SUS) até 60 dias após o diagnóstico ter sido incluído em seu prontuário. Nesse mesmo ano, estimou-se que 11.530 crianças e adolescentes de 0 a 19 anos seriam diagnosticados com câncer, totalizando cerca de 3% do total de casos previstos para aquele ano^(7)^.

Em 2012, o Rio de Janeiro contava com centros especializados em tratamento oncológico, porém não havia uma unidade estadual exclusiva para tratamento onco-hematológico pediátrico. Nesse contexto, deu-se a parceria entre o Governo do Estado e a Organização Social de Saúde (OSS) Instituto D’Or de Gestão de Saúde Pública para criação e gestão de uma unidade estadual que oferecesse esse tipo de atendimento. Assim, foi inaugurado, em março de 2013, no Rio de Janeiro, em Vila Valqueire, o Hospital Estadual da Criança, classificado como de médio porte, com procedimentos de média e alta complexidade^(8)^.

Antes da inauguração do Hospital Estadual da Criança, o espaço abrigava outro hospital privado, que atendia pacientes não oncológicos e de diferentes faixas etárias. Com o contrato com o Estado para abertura de um novo hospital com características diferentes do anterior, foi necessário readequar o quadro de funcionários da enfermagem para atender à nova demanda.

Desse modo, cerca de 70% da equipe de enfermagem foi realocada em outros hospitais compatíveis com as características do hospital que estava em processo de encerramento de suas atividades; 15% tiveram seus contratos encerrados; e 15% da equipe de enfermagem foi absorvida pela nova administração. Desses, em sua maioria, encontravam-se as enfermeiras que tinham cargos de liderança, tais como coordenadoras de enfermagem.

Foram necessárias providências para a reorganização da gerência e das coordenações de enfermagem, de modo a dar conta da implantação do serviço de enfermagem em onco-hematologia pediátrica e da gestão do cuidado, cujas especificidades eram desconhecidas pela maior parte da equipe. Tais providências eram urgentes e necessárias para ocupações de espaços de liderança pela enfermagem.

Nesse mister, as enfermeiras que permaneceram no hospital iniciaram o recrutamento de enfermeiros para o novo espaço hospitalar, ao mesmo tempo em que investiram na formulação de estratégias visando à incorporação de conhecimentos, com o objetivo de dar conta dos desafios impostos em um cenário altamente especializado de cuidado à criança.

A situação histórica apresentada suscitou, portanto, as seguintes questões investigativas: qual a configuração do serviço de enfermagem do hospital no setor de onco-hematologia à época da inauguração? Quais as estratégias empreendidas pelas enfermeiras para ocupar posições de liderança nos espaços do hospital?

## OBJETIVO

Analisar como se deu o processo de implantação do serviço de enfermagem em onco-hematologia de um hospital pediátrico no Rio de Janeiro.

## MÉTODOS

### Aspectos éticos

O estudo foi conduzido de acordo com as diretrizes éticas nacionais e internacionais, aprovado pelo Comitê de Ética e Pesquisa da Escola de Enfermagem Anna Nery/Universidade Federal do Rio de Janeiro, cujo parecer está anexado à presente submissão. O consentimento foi obtido de todos os indivíduos envolvidos no estudo por meio de assinatura presencial do Termo de Consentimento Livre e Esclarecido. O anonimato foi garantido por meio de codificação dos depoimentos e confidencialidade dos dados, guardando os dados em *Google Drive* de uma das pesquisadoras. O estudo foi aprovado sob Parecer n°5.521.434 e Certificado de Apresentação para Apreciação Ética (CAAE) n° 58705822.6.0000.5238 em 11 de julho de 2022.

### Tipo de estudo

Estudo histórico, de abordagem qualitativa. Para seu desenvolvimento, foram seguidos os critérios da lista de verificação do *COnsolidated criteria for REporting Qualitative research* (COREQ).

### Procedimentos metodológicos

As fontes históricas diretas do estudo são documentos escritos e orais. Os critérios de inclusão das participantes foram ser enfermeiras que atuaram na implantação do serviço de enfermagem no recorte temporal do estudo em apreço, ou seja, o período entre 2013-2016, no qual o marco inicial corresponde ao ano em que foi inaugurado o Hospital Estadual da Criança e constituída a equipe de enfermagem, e o marco final representa o ano em que o hospital foi credenciado como Unidade de Alta Complexidade em Oncologia (UNACON). A utilização desses documentos, neste estudo, se justifica no entendimento de que eles fornecem as informações objetivas concernentes ao fenômeno estudado. Nesse propósito, foi necessário triangular os documentos escritos e orais com o contexto, consoantes com uma análise histórica.

### Cenário do estudo

O recorte espacial do estudo é o Hospital Estadual da Criança, cenário que demarcou o fenômeno estudado. Em sua inauguração, possuía dez leitos de Unidade de Terapia Intensiva (UTI) Neonatal, dez leitos de UTI Pediátrica, 58 leitos de Unidade de Internação e oito poltronas de quimioterapia para seguimento de tratamento ambulatorial para pacientes oncológicos, atendendo crianças de 0 a 19 anos de vida.

### Fontes de dados

Os documentos escritos, localizados no Escritório de Qualidade do hospital, são constituídos por: 40 atas de reunião; nove protocolos assistenciais; oito reportagens do site interno, chamado “intranet”, os quais fazem referências às semanas de enfermagem no período do recorte temporal; e indicadores oncológicos (indicadores numéricos, em números absolutos ou percentuais, elaborados pelas coordenações médica e de enfermagem para apresentação à Secretaria Estadual de Saúde, como determina o Termo de Referência “Gestão de Serviços de Saúde no Hospital Estadual da Criança – Oncologia e Cirurgia, no Estado do Rio de Janeiro, por entidade de direito privado, sem fins lucrativos, qualificada como Organização Social” tendo como objeto a contratação de entidade pública sem fins lucrativos para gerir, operacionalizar e executar os serviços de saúde do Hospital Estadual da Criança – Oncologia e Cirurgia”), que demonstraram a realização de 170 diagnósticos de doenças onco-hematológicas e a administração de 9.181 quimioterapias. Os documentos orais foram produzidos por meio de seis entrevistas semiestruturadas, todas com enfermeiras que participaram da implantação do serviço de enfermagem no Hospital Estadual da Criança. Para este artigo, foram utilizadas quatro entrevistas, e elas estão aqui descritas como C1, C3, C4 e C5.

A constituição do *corpus* documental levou em consideração o critério de adequação, constituído por seis etapas: 1) pertinência: as fontes históricas pertencem ao assunto estudado; 2) suficiência: respondem a todas as dimensões do problema de pesquisa; 3) exaustividade; 4) elegibilidade, sem exclusões de fontes, por limitações do pesquisador; 5) representatividade: representam o universo global; 6) homogeneidade: estão organizadas separadamente, uma vez que cada tipo de fonte (escrita e oral) possui tratamento e análise distintos^(9,10)^.

### Coleta e organização dos dados

A coleta de dados ocorreu no período de 01 de agosto de 2022 a 13 de fevereiro de 2023. Foram realizadas seis entrevistas, e quatro tiveram seus excertos aqui apresentados, em contribuição direta ao estudo. Os quatro colaboradores do estudo, aqui apresentados, foram contatados pessoalmente, e os encontros foram agendados e os locais das entrevistas acertados. As entrevistas foram transcritas e devolvidas aos colaboradores sob a forma de texto escrito para a validação, de modo a conferir a transposição do texto oral ao escrito. As fontes indiretas, constituídas por artigos de periódicos científicos produzidos sobre a temática, consubstanciaram a análise dos achados.

### Análise dos dados

Os achados provenientes das fontes escritas e orais foram organizados, triangulados e analisados de acordo com o método histórico^(11)^. Assim, a análise concentra-se em três pilares: heurística, em que ocorre o recolhimento das fontes de informação que serão necessárias à análise histórica; crítica, para avaliar a validade ou não das fontes; e hermenêutica, que possibilita a averiguação dos dados e informações diante das perguntas da pesquisa. Isto posto, os resultados do estudo são oriundos da intercessão da análise do que está contido nas fontes escritas e orais, matéria-prima do estudo, com as fontes indiretas, com base nos contextos políticos, econômicos, sociais e sanitários em que se insere o fenômeno estudado. A confiabilidade dos dados se deu mediante a crítica externa, para verificar a veracidade das fontes, considerando fatores intrínsecos e extrínsecos, crítica e interna, para avaliar a validade e a coerência a partir da própria fonte. É importante assinalar que a legitimação das fontes documentais é tarefa fundamental nas pesquisas históricas de abordagem qualitativa referentes a um passado próximo, sendo a entrevista um recurso valioso para balizar os achados^(12)^.

## RESULTADOS

No período de outubro de 2010 a outubro de 2012, ocorreram 3.000 internações de crianças por neoplasias por ano somente no município do Rio de Janeiro. Dessas, somente 2.000 foram internadas em hospitais especializados nesse tipo de tratamento^(13)^. Essa escassez de atendimento especializado ensejou a proposta do Instituto D’Or de Gestão de Saúde Pública (OSS qualificada no Estado para gestão de hospitais pediátricos) para contratação de entidade privada pelo Estado do Rio de Janeiro.

Sendo assim, em 2012, o Instituto D’Or de Gestão de Saúde Pública elaborou seu Termo de Referência intitulado “Gestão de Serviços de Saúde no Hospital Estadual da Criança – Oncologia e Cirurgia, no Estado do Rio de Janeiro, por entidade de direito privado, sem fins lucrativos, qualificada como Organização Social” tendo como objeto “a contratação de entidade pública sem fins lucrativos para gerir, operacionalizar e executar os serviços de saúde do Hospital Estadual da Criança – Oncologia e Cirurgia”^(8)^.

Em conformidade com o estabelecido no Termo de Referência, o hospital disponibilizado destinava-se ao:

Recebimento de usuários do SUS, referenciados pela Secretaria Estadual de Saúde do Rio de Janeiro para realização de procedimentos cirúrgicos, tratamento clínico onco-hematológico e internação em enfermaria e leitos de unidade de cuidados intensivos e pós-operatório^(8)^.

Nesse contexto, em 2012, com a elaboração do Termo de Referência para disputa de gestão e, posteriormente, em 2013, com a assinatura do contrato de gestão entre o Estado do Rio de Janeiro e o Instituto D’Or de Gestão de Saúde Pública para gerir um hospital que tem como demanda o atendimento de um público humano específico, fez-se necessário o recrutamento de profissionais gabaritados para dar conta desse público, que necessitava de uma atenção altamente especializada.

Tendo em vista que, antes da inauguração do Hospital Estadual da Criança, o prédio cedido para a Secretaria de Saúde abrigava outro hospital particular, que encerrou suas atividades em 2012 para dar seguimento com a reestruturação para abertura da nova instituição, já havia, nesse antigo hospital, uma equipe de enfermagem contratada que endossava o atendimento de enfermagem. Essa fazia parte do contrato de gestão da Rede D’Or que administrava o antigo hospital, responsável por prestar atendimento ao público adulto. Sendo assim, houve um movimento de escolha, pelos coordenadores, dos funcionários que permaneceriam na gestão nova e os que seriam desligados ou remanejados para outros hospitais do mesmo grupo de gestão. Os discursos a seguir configuram a ratificação dessa movimentação, no qual, a partir da resposta à pergunta “Como se deu a sua entrada no Hospital Estadual da Criança?”, é possível averiguar a seleção a partir de duas colaboradoras do estudo:

*Quando houve a mudança para o Hospital da Criança, algumas pessoas foram mandadas embora, outras foram transferidas para outros hospitais da rede* [Rede D’Or]. (C3)

*Eu já trabalhava no hospital anterior, hospital Rio de Janeiro, era* enfermeira *na emergência, eu não ia ficar no hospital, as pessoas que ficariam já tinham sido escolhidas*. (C4)

O atendimento hospitalar altamente especializado necessita de profissionais dotados de conhecimento, para que possam prestar assistência de qualidade, satisfazendo o cumprimento das demandas dos pares-concorrentes que ocupam posições de liderança nesse espaço. Nesse mister, foi necessária a busca de profissionais de enfermagem que demonstrassem capacidade atender um grupo humano específico que necessitava de cuidados onco-hematológicos.

Sendo assim, a contratação de novos profissionais seguiu requisitos singulares, e, para isso, determinou-se que era necessário possuir experiência em pediatria, oncologia, além de comprovação de cursos de capacitação específicos. Essa estratégia visava dar forma a um grupo com *expertise* para lidar com as minucias da onco-hematologia e fortalecer o serviço de enfermagem que estava se formando, e esse enredo é confirmado por uma das entrevistadas:

*Demos preferência para contratar pessoas com, no mínimo, seis meses de experiência em pediatria. Demos preferência para contratar também enfermeiros especializados em neonatal e pediatria, alguns em oncologia e com algumas capacitações, como, por exemplo, curso de inserção de cateter*. (C1)

Na data da sua inauguração, em 01 de março de 2013, o hospital contava com 46 enfermeiros, assim distribuídos: um gerente de enfermagem; cinco coordenadoras; três enfermeiras diaristas; um enfermeira da Comissão de Controle de Infecção Hospitalar; e dois enfermeiras da educação continuada. Além dessas 12 enfermeiras, todas diaristas, havia também quatro supervisoras de enfermagem e 30 enfermeiros que atuavam em regime de plantão. Entende-se, neste hospital, como enfermeiro diarista aquele que atua diariamente na coordenação da assistência aos pacientes. Esse quantitativo inicialmente deu conta das demandas, pois a abertura dos setores do hospital foi gradativa, como informado por uma das colaboradoras:

*Foi gradativamente, como falei lá no início, para que pudéssemos começar a receber as primeiras crianças dentro do perfil, da especialidade, para depois começar os agendamentos, e aí sim as aberturas das unidades de internação e CTI, porque tanto o CTI como a neonatal começaram com a metade do número de leitos.* (C1)

As matérias jornalísticas à época da inauguração do Hospital Estadual da Criança apontavam para o investimento na qualidade da atenção em oncologia pediátrica no Rio de Janeiro, pois, até a inauguração do hospital, somente o Instituto Nacional do Câncer (INCA) realizava cirurgias para tratamento do câncer em crianças da rede pública^(14)^.

Os 46 enfermeiros contratados inicialmente foram distribuídos nos setores pelas enfermeiras remanescentes, que exerciam posição de liderança e que buscavam, no recém-inaugurado hospital, empreender estratégias para obter reconhecimento profissional. A coordenação de enfermagem trabalhou com o objetivo de instituir protocolos que visavam à segurança do paciente e elaboração de práticas baseadas em evidências científicas consubstanciadas e com vistas ao aprimoramento da assistência^(15,16)^. Os relatos das entrevistadas evidenciam que houve um grande investimento desde a inauguração do hospital, com destaque para o ano de 2014, na elaboração de protocolos que embasassem a assistência de enfermagem.

Os protocolos podem ser entendidos como uma medida para a redução dos riscos atrelados à assistência à saúde e, consequentemente, para a redução dos danos desnecessários que podem ser impostos aos pacientes, aumentando, assim, a sua segurança. Desse modo, é uma estratégia que tem como objetivo impedir a ocorrência de danos preveníveis e, quando não for possível evitá-los, minimizar ao máximo o seu efeito negativo para os pacientes. Isto posto, é imprescindível avaliar quais atitudes realizadas pelos profissionais de saúde fomentam a segurança do paciente, e os protocolos possibilitam essa avaliação^(17,18)^.

Entre os protocolos instituídos, percebe-se a adequação à realidade de cuidados de enfermagem onco-hematológicos, destacando-se protocolo de intervenção diante do derramamento acidental dos quimioterápicos antineoplásicos, protocolo de assistência de enfermagem na prevenção de extravasamento de antineoplásicos e consulta de enfermagem no ambulatório de onco-hematologia. O relato de uma das entrevistadas deixa claro o entendimento da importância dos protocolos para a assistência segura:

*Participamos da produção desses protocolos novos com a gerência de enfermagem*. [...] *e a gente ia trazendo contribuições conforme ia estudando e montamos esses protocolos*. (C5)

Paralelamente à elaboração de protocolos, foram criadas as comissões hospitalares, responsáveis por avaliar a efetividade e a qualidade do serviço assistencial, além de discutir casos específicos, elaborar pareceres, propor iniciativas de melhorias, trazer sugestões de melhorias do serviço e avaliar e elaborar indicadores que demonstrassem a qualidade da assistência. Os membros dessas comissões eram indicados e nomeados pela diretora geral e diretor médico do hospital. Uma das comissões mais importantes e de presidência de uma enfermeira foi a Comissão de Cateteres Venosos Centrais, que tinha como objetivo proceder com a regulamentação dos dispositivos venosos do hospital, fornecer dados estatísticos e promover o desenvolvimento técnico-científico da equipe de enfermagem.

Durante as entrevistas, as colaboradoras do estudo relataram que, posteriormente às redistribuições de cargos e alocações dos enfermeiros diaristas, no entendimento de trabalhar a educação em saúde de forma ampliada, as coordenadoras de enfermagem tinham como meta reproduzir os conhecimentos em onco-hematologia, por elas adquiridos nos treinamentos, cursos de pós-graduação e visitas às unidades especializadas, para a equipe de enfermagem do hospital.

As estratégias, no sentido de buscar aprimoramento profissional, podem ser observadas quando, dois meses após a inauguração do hospital, em maio de 2013, as enfermeiras já realizaram a primeira Semana da Enfermagem. A programação reforçava a preocupação em abordar temas relacionados à onco-hematologia e aos cuidados necessários, tais como biossegurança em quimioterapia (ministrado por palestrante do INCA), intercorrências clínicas em oncologia (palestrante docente do curso de pós-graduação de oncologia clínica), gerenciamento de enfermagem no setor de quimioterapia (palestrante enfermeiro do Hospital Pedro Ernesto). Como se pode constatar, houve a preocupação em trazer enfermeiros de hospitais de referência, como o INCA, e hospitais de grande porte, como o Hospital Pedro Ernesto, para ministrar palestras.

Em cumprimento das obrigações contratuais, no que concerne à prestação de contas, constantes no Termo de Referência, o hospital precisou elaborar indicadores que demonstrassem a efetividade e a qualidade da assistência prestada. Assim, as enfermeiras tiveram que atender à solicitação de elaboração de tais indicadores. Entre os indicadores elaborados, avaliados e acompanhados, relacionados à onco-hematologia, estavam número de quimioterapias administradas, número de quimioterapias vesicantes, número de derramamentos de quimioterápicos, número de extravasamento de quimioterápicos e número de lesões por extravasamento. Esses indicadores revelariam diretamente a qualidade assistencial da enfermagem, porque são atividades exclusivas desses profissionais. Apesar de não serem feitas diretamente pelas coordenações ou gerência, os resultados refletiriam suas gestões, já que as atividades são baseadas nos protocolos elaborados e treinamentos realizados por essas. Esse feito fez com que as enfermeiras fossem conhecidas e reconhecidas pelos pares, concorrentes ou superiores no interior da instituição.

Além disso, algumas das matérias midiáticas relacionadas ao hospital referiam-se ao fato de que o Hospital Estadual da Criança tinha recebido o título de acreditação pela Organização Nacional de Acreditação (ONA), desde 2014, sendo certificado com excelência desde 2015. A ONA é uma entidade que, desde 1999, trabalha em prol da adoção, pelas instituições de saúde, de uma gestão e assistência que leva à melhoria do cuidado e segurança do paciente.

A segurança do paciente em unidades hospitalares fomenta discussões no âmbito nacional e internacional. Diante disso, a Organização Mundial da Saúde (OMS) criou um programa, em 2004, denominado “Aliança Mundial para a Segurança do Paciente” (*World Alliance for Patient Safety*), tendo como objetivo avaliar a segurança do paciente nos serviços de saúde. A segurança do paciente é uma questão de saúde pública que reverbera em todo mundo e necessita de planos universais e eficazes. A insegurança e os danos causados aos pacientes são as principais causas de doenças globais e que têm como principais causas evitáveis os erros de administração de medicação e práticas inseguras^(19)^.

Além disso, anualmente, ocorrem 134 milhões de eventos adversos em hospitais de países considerados de baixa ou média renda, resultando em 2,6 milhões de mortes, devido a esses cuidados inseguros^(20)^. Apesar dos números alarmantes que envolvem países de baixa e média renda, países considerados desenvolvidos e centros do mundo, como Estados Unidos da América e Reino Unido, não estão completamente a salvo dos erros relacionados à assistência insegura, refletindo um incidente com dano a cada 35 segundos no Reino Unido e sendo a terceira causa de óbito nos Estados Unidos da América^(21)^.

No Brasil, as discussões relacionadas à segurança do paciente acontecem desde 2002, com a criação da Rede Brasileira de Hospitais Sentinela pela Agência Nacional de Vigilância Sanitária (ANVISA), porém, somente em 2013, houve a instituição do Programa Nacional de Segurança do Paciente (PSP) pelo Ministério da Saúde, através da Portaria GM nº 529, tendo como objetivo central contribuir para qualificação do cuidado em saúde^(22)^. Desde a instituição do PSP até o ano de 2022, houve 1.100.352 incidentes notificados, mas acredita-se que esse número ainda possa ser maior, devido a subnotificações^(23)^.

O potencial de risco ou dano ao paciente é inerente à área da saúde, e a busca contínua pela minimização dos danos é imprescindível. A OMS revela que, para um atendimento ser considerado de qualidade, é necessário haver aceitabilidade, equidade, segurança, eficiência e eficácia^(24)^. Traz ainda a recomendação em seu documento intitulado “*Patient safety : making health care safer*”^(25)^ que os estabelecimentos de saúde desenvolvam suas atividades embasados em acreditações e evidências e práticas científicas.

Sendo assim, a partir dos dados de indicadores apresentados para a Secretaria de Saúde do Estado do Rio de Janeiro, pela incorporação de um certificado de importância nacional, como a acreditação pela ONA, apresentação de parâmetros de assistência específicos e após apresentar recursos humanos em consonância com os padrões estabelecidos, condições técnicas, infraestrutura exclusiva e equipamentos e demonstrar um cuidado especializado em oncologia pediátrica e hematologia oncológica de crianças e adolescentes, o Hospital Estadual da Criança pôde ser habilitado como UNACON em 2016.

De acordo com o INCA (2016), em 2006, havia 285 estabelecimentos de saúde habilitados para atendimento em oncologia vinculados ao SUS, e somente 71 eram habilitados para oncologia pediátrica, ou seja, esses locais perfaziam um total de menos de 25%. Na região Sudeste, havia 136 serviços habilitados em oncologia e 35 em oncologia pediátrica, sendo que, desses, apenas cinco encontravam-se no estado do Rio de Janeiro. De acordo com a Portaria nº 140 de 2014, o município do Rio de Janeiro tinha 13 estabelecimentos habilitados para oncologia, porém os estabelecimentos habilitados para tratamento de oncologia e hematologia pediátrica seguiam sendo cinco, e somente uma instituição federal era cadastrada como UNACON exclusiva de pediatria^(13)^.

A inauguração do Hospital Estadual da Criança em 2013 trouxe para o estado do Rio de Janeiro a possibilidade de diagnóstico e tratamento onco-hematológico e diagnóstico de outras doenças hematológicas com um atendimento exclusivo para o público infanto-juvenil. A parcela dessa população que era atendida, muitas vezes, em centros não especializados ou em unidades mistas, e que demorava longos períodos nas filas da regulação, pôde deixar de peregrinar e iniciar os trâmites para diagnóstico e tratamento em um local com qualidade de assistência e especificidade na onco-hematologia pediátrica, com instalações exclusivas, acesso a todos os medicamentos e equipamentos necessários para diagnóstico-terapias e recursos humanos compatíveis com o cuidado específico que esse público exige.

Além disso, a demanda reprimida que havia em 2012 ganhou um hospital capaz de absorver e poder, até certo ponto, alcançar a tríade de países desenvolvidos para o sucesso do tratamento, isto é, equipe qualificada, acesso rápido e de qualidade a diagnósticos e tratamento e acessibilidade a medicamentos. No seu ano de inauguração, a instituição diagnosticou 46 doenças onco-hematológicas e realizou 1.175 quimioterapias; em 2014, foram 40 doenças onco-hematológicas e 3.083 quimioterapias; em 2015, ofertou 39 diagnósticos e realizou 2.389 quimioterapias; e no último ano do estudo, em 2016, 45 crianças e adolescentes tiveram seus diagnósticos e 2.534 quimioterapias foram administradas.

Entre 2013 e 2016, o Hospital Estadual da Criança forneceu 170 diagnósticos de doenças onco-hematológicas malignas e 263 diagnósticos de doenças onco-hematológicas benignas, mas que necessitavam de acompanhamento com especialista. Isso significa que 433 crianças e adolescentes puderam receber atenção e cuidados especializados e não foram expostos a instituições que não possuem equipes com *expertise* na área. Além disso, no mesmo período, foram realizadas 9.181 administrações de quimioterapias.

No que concerne às quimioterapias, essas são realizadas exclusivamente por enfermeiros capacitados e que possuem habilidades para lidar com as intercorrências clínicas que podem acontecer ao longo das administrações e que necessitam que sejam traçados planos para minimizar os riscos ao paciente. O gerenciamento desses riscos, de forma individual e pautado em conceitos técnicos e científicos, bem como os números de indicadores gerados a partir desse atendimento, que demonstram a boa assistência de enfermagem, como não haver lesão por extravasamento de quimioterápicos durante todo o recorte temporal, ministração de cursos específicos na área e a constante evolução de protocolo, certamente foram condições avaliadas para que a habilitação do Hospital Estadual da Criança como UNACON fosse concedida, como disposto na Portaria nº 2.491, de 28 de dezembro de 2016.

## DISCUSSÃO

No Rio de Janeiro, em 2012, havia uma demanda reprimida de crianças e adolescentes carentes de atendimento onco-hematológico, devido à falta de hospitais especializados para esse tipo de assistência^(13)^. Com o intuito de administrar essa questão, ocorreu uma parceria entre o governo do Estado do Rio de Janeiro e uma OSS, o Instituto D’Or, para inaugurar um hospital público que absorvesse essa clientela para tratamento. Devido ao investimento, o Instituto D’Or necessitou adquirir mão de obra especializada que desse conta desse serviço específico.

As OSS são entidades que estão associadas ao terceiro setor. Juridicamente são entidades sem fins lucrativos, porém com características privadas. O terceiro setor traz um modelo de gestão que, apesar de dar suporte a diversas demandas de assistência social, “se oferece como alternativa para a transferência das responsabilidades sociais do Estado. O governo assume o papel de regulador e as instituições não governamentais apoiam e substituem o Estado”^(26)^.

Para desenvolver um cuidado universal, integral e com equidade, princípios do SUS, é necessário gerenciar a instituição de modo eficiente e com qualidade. Para isso, os gestores necessitam organizar o serviço, de modo a garantir a atuação da equipe de enfermagem de forma resoluta, eficiente, que proporcione satisfação aos clientes e, ao mesmo tempo, motive e dê legalidade à sua equipe^(27,28)^.

Sendo assim, referente às estratégias para implantar o serviço de enfermagem em 2012, quando houve a dissolução do Hospital Rio de Janeiro para reformulação do que viria ser o Hospital Estadual da Criança, as enfermeiras remanescentes que permaneceram com a função de estruturar esse novo serviço superaram a falta de conhecimentos técnicos em onco-hematologia pediátrica através da incorporação de conhecimentos técnico-científicos, necessários para manter suas posições dentro da instituição. Elas buscaram esses conhecimentos em instituições de alto padrão, reconhecidas pelos seus trabalhos na onco-hematologia pediátrica, além da participação em cursos e em pós-graduações que lhes conferissem certificações legitimadas que pudessem ratificar a inclusão desse novo conhecimento, iniciando, aí, o movimento efetivo de implantação do serviço.

Trata-se de uma estratégia eficaz requerida para aumentar o conhecimento destas enfermeiras. Estudo com 1.312 enfermeiros em cinco hospitais no Irã, sobre desenvolvimento de modelo de acreditação, evidenciou que o investimento no desenvolvimento profissional impacta a melhoria dos serviços prestados e que essas iniciativas colaboram para o alcance da acreditação, o que ocorreu com o referido hospital através da busca das enfermeiras pelas suas qualificações^(29)^.

Posteriormente à busca desses conhecimentos, houve a contratação de pessoal qualificado nos termos da gestão, ou seja, portadores de conhecimentos especializados compatíveis com as demandas vindouras nesse espaço. Assim, os requisitos foram ter experiência em onco-hematologia, pediatria e apresentar cursos específicos na área - condição *sine qua non* para implantação do serviço. A qualificação desses profissionais contratados para desenvolver o serviço dentro do Hospital Estadual da Criança ocorreu através da replicação dos conhecimentos adquiridos pelas enfermeiras remanescentes por meio de treinamentos e práticas direcionadas.

Revisão integrativa recente evidenciou que o enfermeiro desenvolve, em sua prática profissional, o papel de educador, promovendo a educação continuada, desenvolvendo ações direcionadas para ensinamentos de autocuidado ao paciente, além de oferecer treinamentos e contribuir para a disseminação da cultura da qualidade. Além disso, busca conhecimento através de desenvolvimento de pesquisas e discussão de casos clínicos^(29)^. Nesse sentido, a qualificação dos profissionais do Hospital Estadual da Criança foi essencial para o êxito da implantação do serviço, e desponta como uma das funções essenciais do enfermeiro em processos de mudanças no trabalho, visando a uma melhor prestação de serviços. A pesquisa destaca, ainda, a importância de os gestores incentivarem e alocarem recursos para a efetivação das ações de educação e treinamento nas instituições.

A escolha das enfermeiras que permaneceriam no novo hospital evidencia cabalmente que elas foram preservadas nos seus cargos pelo reconhecimento dos gestores do hospital das suas qualidades técnicas, que possibilitariam o desenvolvimento de suas coordenações através da busca e estratégias de capacitação e reestruturação do serviço. Sendo assim, foi dada a elas a incumbência de formação da equipe de enfermagem que daria base para aquela nova realidade.

Essas coordenadoras de enfermagem, em face da autoridade que exerciam no antigo hospital, foram consideradas detentoras de competência profissional para promover o recrutamento das enfermeiras que integrariam a nova instituição. Essa condição também pode ser explicada pela compatibilidade necessária entre as demandas da nova experiência com aquelas já adquiridas, que as colocavam em outro patamar diante dos gestores.

No que concerne ao número de profissionais de enfermagem, vale ressaltar que, entre 2013 e 2018, houve um aumento de 4,7 milhões no mundo, porém, apesar desse aumento, ainda existe uma escassez estimada em 5,9 milhões de profissionais. Coadunado a isso, a OMS publicou, em 2020, um relatório intitulado *“State of the World’s Nursing 2020: Investing in Education, Jobs and Leadership”* ^(30)^, o qual destacou o papel fundamental da enfermagem na promoção da saúde e prevenção de doenças. A enfermagem é a maior categoria de profissional de saúde em todo o mundo, representando 59% da força de trabalho em saúde.

O referido relatório destaca ainda que a maior parte desses enfermeiros trabalha em países de baixa ou média renda e que enfrentam desafios significativos, incluindo condições inadequadas para realizar seus trabalhos. Traz ainda como ponto fundamental a demanda de investir em lideranças, de modo que os enfermeiros cumpram papel ativo nas tomadas de decisões e desenvolvimento de estratégias em saúde.

Isto posto, pôde ser percebido que as enfermeiras do Hospital Estadual da Criança implantaram um serviço de enfermagem de forma consoantes com as demandas de saúde, garantindo assim a qualidade do serviço prestado e fomentando a relevância da força de trabalho da enfermagem em um cenário altamente especializado.

A parceria com instituições já reconhecidas nacionalmente, no que se refere ao setor educacional, transformou as estratégias de elaboração do serviço através da incorporação de conhecimentos sobre o gerenciamento dos cuidados de enfermagem em pacientes pediátricos que necessitavam de atenção especializada. Outrossim, a capacitação da enfermagem permite que a realização dos processos ocorra em consonância com as normas, a fim de garantir a segurança do paciente através da educação desses profissionais, sendo uma ferramenta de continuidade do cuidado seguro^(31)^.

Revisão sistemática, realizada entre 1999 e 2021, para sintetizar e avaliar o efeito cumulativo da intervenção educativa sobre segurança do paciente para profissionais de saúde no ambiente hospitalar em sua cultura de segurança do paciente, evidenciou que programas de educação para este fim podem melhorar a cultura da segurança entre profissionais de saúde e pacientes, o que corrobora as estratégias desenvolvidas pelas enfermeiras do Hospital Estadual da Criança^(32)^.

Essa busca pela incorporação de conhecimentos pela equipe de enfermagem do hospital, com o propósito de consolidação de um serviço de enfermagem em onco-hematologia pediátrica com qualidade, está em consonância com a política da OMS no Plano de Ação Global para a Segurança do Paciente 2021-2030, em que nenhum paciente pode ser vítima de eventos adversos, devendo receber sempre cuidado seguro e respeitoso em todos os locais e etapas dos atendimentos^(33)^. Melhorar a segurança do paciente é um objetivo político na área da saúde em todo o mundo^(34)^.

Ademais, a segurança do paciente, que requer avaliações contínuas e manutenção de uma cultura permanente de educação continuada, baseada na realização de cursos de aperfeiçoamentos, institucionalizações de protocolos, indicadores e comissões científicas de enfermagem, trouxe gabarito suficiente para que houvesse avaliação e certificação do Hospital Estadual da Criança pela ONA e que fosse conferido a ele acreditação por excelência^(35)^. Isso porque a segurança do paciente representa um dos grandes desafios para as organizações de saúde do século XXI e, portanto, requer prestação de serviços de qualidade^(35)^.

Pesquisas recentes destacam que uma cultura de segurança pode ser estabelecida por meio de discussões sobre erros, eventos adversos e seus impactos para a qualidade do cuidado. Destacam também que programas de formação são necessários para criar uma cultura de participação dos pacientes na gestão de seu próprio cuidado e melhorias da capacidade de identificação de incidentes de segurança do paciente^(36,37,38)^.

Revisão integrativa sobre atuação do enfermeiro em um contexto de acreditação hospitalar apontou que o enfermeiro é o protagonista na busca pela certificação, pois possui atribuições na assistência, na administração e na educação, os quais são fundamentais para a conquista do selo de qualidade. Destaca também que o credenciamento incentiva práticas de educação continuada através de treinamentos dos profissionais de saúde com o objetivo de promoção das melhores práticas e desenvolvimento de perfis de profissionais^(39)^. Nesse sentido, devido ao credenciamento, as enfermeiras utilizaram suas habilidades de liderança, capacidade de transmissão do conhecimento e facilidade para o trabalho em equipe, o que contribui para o êxito da implantação.

Em 2023, o Hospital Estadual da Criança completou sua primeira década de funcionamento e, em comemoração, realizou um simpósio intitulado “Uma nova realidade da Saúde Pública no Rio de Janeiro”, demonstrando o resultado de dez anos de trabalho e retorno à sociedade. Contando com 56 leitos de unidade de internação ativos, dez leitos de UTI Pediátrica e dez leitos de UTI Neonatal, tendo iniciado suas atividades em 2013 com 46 enfermeiros, chegando a 101 em 2023, realizou mais de 200 mil consultas ambulatoriais em todas as suas especialidades médicas e mais de 22 mil administrações de quimioterapia, realizadas pelos enfermeiros, para os pacientes oncológicos, possibilitando entregar à sociedade qualidade de vida para essas crianças e adolescentes, além de desenvolvimento social, econômico e pessoal.

Além disso, é cabal que a enfermagem é a espinha dorsal do SUS, e o seu trabalho pode ser percebido em ações sistematizadas que visavam assistência integral à sociedade. Estar entre apenas alguns hospitais públicos do Brasil certificados pela ONA, título que foi conquistado com a participação efetiva das enfermeiras, traz à tona o peso que a implantação e a consolidação do serviço de enfermagem tiveram para além da instituição, atingindo camadas da sociedade que, em outro momento, poderiam não ter à sua disposição um serviço de saúde público com o gabarito e as experiências desenvolvidas. Todo esse contexto e a colaboração *sine qua non* do serviço de enfermagem em onco-hematologia pediátrica culminou na habilitação do Hospital Estadual da Criança como a primeira UNACON (estadual) exclusiva de pediatria no Rio de Janeiro.

### Limitações do estudo

Uma limitação do estudo foi a concordância, por parte de algumas colaboradoras, ora em conceder a entrevista, ora em serem localizadas, o que suscitou a busca através de redes sociais e aplicativos de mensagens instantâneas. Sendo assim, houve demora em conseguir realizar as entrevistas e também um menor número de colaboradoras do que o esperado inicialmente. Apesar dessas limitações, as respostas às questões investigativas foram respondidas.

### Contribuição para a área da enfermagem, saúde ou políticas públicas

O estudo demonstra que a inserção do enfermeiro em áreas altamente especializadas, com um serviço de enfermagem pautado em conhecimentos técnico-científicos robustos e uma cultura de segurança disseminada através de protocolos, comissões e indicadores, implica diretamente a qualidade do serviço, sendo reconhecido e ratificado não só pelos pares, mas também por instituições de renome nacional, como a ONA, além de ser habilitado como UNACON exclusiva de pediatria pelo Ministério da Saúde. Isto posto, fomenta o alcance dos princípios do SUS e devolve à sociedade o que se espera: um perfil de serviço de enfermagem diferenciado que expande sua atuação, a fim de impactar positivamente a vida da população.

## CONSIDERAÇÕES FINAIS

O empreendimento de implantar e consolidar um serviço de onco-hematologia pediátrica foi exitoso e permitiu que a equipe de enfermagem capitalizasse reconhecimento social de sua importância na equipe de saúde do hospital.

As enfermeiras foram elementos-chave nesse processo, ao apresentarem um papel assistencial, gerencial, educador e político. Os protocolos, formulações de indicadores, comissões e eventos científicos, ao mesmo tempo em que propiciaram segurança aos pacientes, também foram de grande valia para que o hospital fosse habilitado como UNACON exclusiva de pediatria, conferido pelo Ministério da Saúde, atingindo, assim, a proposta de entregar à sociedade um serviço de qualidade técnica, científica e humana, contribuindo para o fortalecimento do SUS.
